# A Review of Sports-Related, Life-Threatening Injuries Presenting to Emergency Departments, 2009–18

**DOI:** 10.5811/westjem.18630

**Published:** 2025-02-24

**Authors:** Abiye Ibiebele, Rebekah Mannix, William Meehan

**Affiliations:** * Massachusetts General Hospital, Department of Emergency Medicine, Boston, Massachusetts; † Massachusetts General Hospital, Department of Orthopaedic Surgery, Boston, Massachusetts; ‡ Boston Children’s Hospital, Department of Emergency Medicine, Boston, Massachusetts; § Boston Children’s Hospital, Division of Sports Medicine, Micheli Center for Sports Injury Prevention, Boston, Massachusetts

## Abstract

**Introduction:**

In the United States, 3.7 million people present to an emergency department (ED) annually with an injury related to sports or athletic activity. A prior study a decade ago revealed that 14% of life-threatening injuries presenting to EDs were sports related, with this percentage being higher in the pediatric population. However, with changes in sports participation and regulatory changes over the past decade, it is unclear whether the proportion of life-threatening sports-related injuries has changed.

**Methods:**

We conducted a cross-sectional study using the National Hospital Ambulatory Medical Care Survey (NHAMCS), consisting of patients from years 2009–2018. Life-threatening injuries were defined as International Classification of Diseases 9 and 10 codes for skull fracture, cervical spine fractures, intracranial hemorrhage, traumatic pneumothorax/hemothorax, liver lacerations, spleen lacerations, traumatic aortic aneurysm or rupture, gastric/duodenal rupture, heat stroke, and commotio cordis. Injuries were classified as sports related based on external cause of injury codes. We examined the relationship between demographic variables and sports-related injuries using Pearson chi-square analysis.

**Results:**

From the years 2009–2018 there were 256,564 observed ED visits. Of these, 646 were for life-threatening injuries, representing a national estimate of 3,456,166 patients over the 10-year period. Thirteen percent were sports related. Of the life-threatening injuries, 77.5% were injuries to the head and neck, and 9.1% of these were sports related. The proportion of life-threatening injuries due to sports and recreation was higher among pediatric patients than adult patients (30.4% vs 9.9%, P<0.001). The proportion of sports-related life-threatening injuries to the head and neck was also higher among pediatric patients than adult patients (23.3% vs 6.4%, P<0.001)

**Conclusion:**

A substantial proportion of life-threatening injuries occur during sports and recreation, especially among pediatric patients. Compared to a similar study a decade ago, there is a similar proportion of life-threatening injuries that are sports related, however; there does seem to be a decrease in the proportion of life-threatening sports-related injuries to the head and neck. Sports medicine physicians and sports organizations should continue to find effective ways to prevent life-threatening injuries in sports.

## INTRODUCTION

In the United States, approximately 3.7 million people present to an emergency department (ED) annually with an injury related to sports or athletic activity.^1^ Over two-thirds of these injuries are in patients 5–24 years of age.^1^ From 2010–2016, this age group alone had 2.7 million ED visits due to injuries related to sports and athletic activity.^2^ Football, basketball, pedal cycling, soccer, and skating/skateboarding led to the most ED visits for sports-related injury within this age group.^1^,^2^ According to a prior analysis of life-threatening injuries in sport using the National Hospital Ambulatory Medical Care Survey (NHAMCS) 1999–2008, 14% of all life-threatening injuries presenting to EDs and ambulatory care centers in the US were sports related.^3^ In pediatric patients, this number increased to 32%, compared to 9% in adults.^3^

Over the last decade there have been developments in sports that may serve to decrease the percentage of sports-related life-threatening injuries. According to the National Federation of State High School Associations, participation in high school football has decreased from 2009–2018.^4^ In addition, various rule changes have been implemented in the past decade to make sports safer. For example, youth ice hockey in some areas has increased the age limit of when body checking is allowed from 11 to 13 years of age, which has decreased the overall number of injuries.^5^ Furthermore, the use of safety equipment such as bicycle helmets has increased over time.^6^,^7^ We used the NHAMCS database to describe the proportion of all life-threatening injuries presenting to EDs in the US that are related to sports or athletic activity.

## METHODS

We conducted a cross-sectional study using the NHAMCS database, focusing on the years 2009–2018.^8^ The NHAMCS is an annual survey of hospital emergency and outpatient departments designed by the National Center for Health Statistics. It is designed to gather data on utilization and provision of ambulatory care services. Every year, a nationally representative sample is created representing visits to EDs in non-institutional general and short-stay hospitals, exclusive of federal, military, and Veterans Administration hospitals, located in the 50 states and the District of Columbia. This survey uses a three-stage probability sampling design. Data collected in the sample consists of approximately 25,000 visits to approximately 500 hospitals; these visits are then weighted by the survey staff, which are then used to derive national estimates.

Data is collected from hospitals during a randomly selected four-week reporting period. Trained interviewers collect data using a standardized patient record form. The NHAMCS dataset is publicly available via the internet. This study was approved by our institutional review board.

The inclusion criteria were patients seen in an ED and diagnosed with life-threatening injuries. Our focus was the 10-year period of 2009–2018, during which NHAMCS changed from International Classification of Diseases Rev 9 (ICD-9) to ICD-10 in 2016. We defined life-threatening injuries using ICD-9/ICD-10 codes for skull fracture (ICD-9 800.x-801.xx, 803.x-804.xx/ ICD-10 S02.0x, S02.1x); cervical spine fractures [ICD-9 805.xx-805.1x, 806.1x/ ICD-10 S12.0x-S12.7x, S12.9x]; intracranial hemorrhage (ICD-9 852.xx-853.xx/ ICD-10 S06.3x-S06.9x); traumatic pneumothorax/hemothorax (ICD-9 860.00–860.50/ ICD-10 S27.0x-S27.2x); liver lacerations [ICD-9 864.xx/ ICD-10 S36.11x]; spleen lacerations [ICD-9 865.xx/ ICD-10 S36.03x]; traumatic aortic aneurysm or rupture (ICD-9 901.0, 902.0/ ICD-10 S25.0x, S35.0x); gastric/duodenal rupture (ICD-9 537.89/ ICD-10 S36.3x, S36.4x); heat stroke (ICD-9 992.0/ ICD-10 T67.0x); and commotio cordis [ICD-9 861.01/ ICD-10 S26.11, S26.91)—all of which were used for a similar analysis examining the prior decade.^3^ We excluded some diagnosis codes included in the prior study, specifically codes ICD-9 802.xx, as these represent facial fractures, which are unlikely to represent life-threatening injuries. In addition, we excluded all 805.xx codes after 805.1x as these injuries represented spinal fractures not localized to the cervical spine and, therefore, are also unlikely to represent life-threatening injuries.

We characterized life-threatening injuries as sports related using external cause of injury codes (E-codes), noted in the Appendix. We only included E-codes that were unique to sports and athletic activity, as described in Rui et al.^2^ In prior studies researchers could conduct a verbatim text search of the NHAMCS dataset in addition to using the E-code to confirm that an E-code truly represented a sports-related cause of injury, as well as further capture sports-related injuries that may not have been captured by coding.^2^,^3^ However, after 2009 the NHAMCS no longer included a verbatim cause of injury for patients in their public use database. Thus, we were unable to perform a text search on E-codes that may have been sports related, but the injury itself was not unique to sports (eg, ICD-9 E880-E888; accidental fall). To keep our estimate conservative, we did not categorize such E-codes as sports related.

We examined demographic data (age, sex race, geographic location) as described in the NHAMCS dataset. All patients >18 of age were categorized as adults. Patients were further characterized into these age groups: preschool (0–5 years); school aged (6–18); young adult (19–44); middle-aged adults (45–74); and elderly (75 and older). We used weights, strata, and primary sampling unit design variables provided by NHAMCS for all analyses. We used descriptive statistics, with appropriate weighting to account for survey sampling methodology, using SPSS Statistics version 29 (IBM Corp, Armonk, NY). The relationship between demographic variables and sports-related injuries was examined using Pearson chi-square analysis.

## RESULTS

From 2009–2018, there were a total of 256,564 observed ED visits in the NHAMCS database. Of these, 646 represented life-threatening injuries which, when accounting for weighting, yields an estimate of 3,456,166 patients with life-threatening injuries over the 10-year period. Of the life-threatening injuries, 13.0% were sports related, representing an estimated 449,957 patients nationally over the time period. A higher proportion of life-threatening injuries were sports related in the pediatric population compared to adults (30.4% vs 9.9%, *P*<0.001 (see Table). The highest proportion of sports-related life-threatening injuries was observed in patients 6–18 years old; however, patients 19–44 years of age accounted for the highest absolute number of sports-related life-threatening injuries (Table).

Head and neck injuries made up 77.5% of the life-threatening injuries. Of the head and neck injuries, 9.1% were sports related, representing a national estimate of 243,387 patients. Among pediatric patients, a higher proportion of life-threatening head and neck injuries were sports related when compared to adults (23.3% vs 6.4%, *P*<0.001).

## DISCUSSION

Approximately 1 in 7 life-threatening injuries presenting to EDs in the US are related to sports and athletic activity. Children have a higher percentage of sports-related life-threatening injuries compared to adults. Our main purpose in this study was to determine whether the prevalence of sports-related life-threatening injuries has changed in the last decade. When comparing to a similar study using NHAMCS 1999–2008, we found similar rates of sport-related life-threatening injuries.^3^

Almost 80% of the life-threatening injuries in this cohort were injuries to the head and neck. Of these injuries, 1 of 10 were sports related. The 38^th^ annual report from the National Center for Catastrophic Injury notes that injuries to the head/brain and spine account for a high proportion of traumatic catastrophic sports injuries.^9^ However, when comparing to the 2013 study, we found a lower percentage of sports-related life-threatening injuries of the head and neck.^3^ In our cohort, this percentage is 9.1%; this has been reported in the past in a similar study to be 14%.

Children had a higher percentage of sports-related head and neck life-threatening injuries than adults. This trend has also been described recently in a study of emergency medical services activations for sports-related injuries in 2017–2018.^10^ This trend may reflect a higher risk of injury during sports participation for children than adults; however, it is also possible that a higher proportion of children participate in sports and athletic activities than adults, thus accounting for the findings. We could not determine the reasons for this discrepancy using the NHAMCS dataset as the number of participants in sports and recreational activities is unknown.

The results of this study suggest that even though the percentage of life-threatening injuries to the head and neck has decreased, there remains a continued need to focus on decreasing the prevalence of these injuries, especially in the pediatric population. Further research efforts should focus on continuing to collect data on these injuries in all age groups and sports levels to identify trends and patterns in injury occurrence. Also, while rule changes have helped to make sports safer, a continued commitment to sportsmanship and vigilance in preventing flagrant rule offenses, as well as injury prevention programs such as training athletes how to fall safely and practice neuromuscular control, have shown promise in preventing injury.^5^,^11^,^12^

## LIMITATIONS

Although we used a similar study from a decade ago to compare the prevalence of sports-related life-threatening injuries, this study was not a direct comparison between the two datasets given differences in coding and included data. Like the prior dataset, we did include ICD codes for intracranial hemorrhage, including the ICD-10 code, S06.9. This code does describe injuries coded as unspecified intracranial injuries; so it is possible that some of these injuries may represent concussions. However, given that we excluded the code specific for concussions, (S06.0 codes), we felt a majority of these codes would not represent concussions. In addition, there are some limitations in the capture of the injuries classified as sports related. As mentioned previously, the NHAMCS no longer included a verbatim cause of injury in their public use database after 2009, precluding us from capturing sports-related injuries that were not captured using the E-codes alone. Additionally, the care team at the time of initial ED presentation may not have coded all activities related to sports and athletics.

While it has not been investigated in the NHAMCS dataset, there may be an initial coding bias with regard to external cause of injuries, as a youth athlete presenting from an organized sport may be more likely to receive a sports-related, external cause of injury code than an older patient who is more likely to participate in individual athletic activity instead of an organized sport. Given that this study is an analysis of a publicly available dataset, it does have the caveat that some initial presentations may have been miscoded initially. The NHAMCS dataset was developed specifically to estimate the incidence of injuries and illness and has been used numerous times in the literature to this effect; however, our study findings should be interpreted with this in mind. Given that prior analyses of sports-related injuries, including one in the prior decade, also used the NHAMCS dataset, we do not expect miscoding to bias this analysis more than prior analyses described in the literature.

## CONCLUSION

The results of this study suggest that the overall proportion of life-threatening injuries directly related to sports and athletic activities has overall remained steady over the past decade. This data does suggest that with an increased focus on injury prevention over the past decade, sports-related life-threatening head and neck injuries have decreased when compared to the findings in prior literature. This could represent a change in the pattern of injury; further studies should be done to investigate whether this pattern is seen in other datasets and its potential causes. We emphasize that there is still a need for sports medicine physicians, researchers, and sports organizations to continue to find effective ways to prevent these injuries and improve the safety of sports for all participants.

## Supplementary Information



## Figures and Tables

**Table:** Proportion of life-threatening injuries related to sports from 2009–2018.

Demographic	Total life-threatening injuries	Sports related-injuries (weighted %)*	National estimate (in thousands)	P-value
Sex				
Female	237	12 (7.5%)	94	<.001
Male	409	52 (16.2%)	356	
Age				
Adult	540	37 (9.9%)	288	<.001
Pediatric	106	27 (30.4%)	162	
Age group				
Under 6 years of age	45	1 (8.7%)	19†	<.001
6 to 18 years of age	61	26 (45.9%)	142	
19 to 44 years of age	174	20 (18.1%)	165	
45 to 74 years of age	206	15 (10.1%)	114	
≥75 years of age	160	2 (1.0%)	8†	
Race				
Black	68	5 (7.4%)	22†	<.001
White	538	56 (14.0%)	416	
Other	40	3 (6.5%)	12†	
Region				
Midwest	156	20 (16.8%)	142	<.001
Northeast	144	13 (6.4%)	42	
South	202	14 (11.1%)	134	
West	144	17 (18.0%)	131	

* This column demonstrates the number of cases in the dataset per demographic group that were classified as sports related. Using weights provided by the National Hospital Ambulatory Medical Care Survey to calculate national estimates, weighted percentages were obtained.

† Given lower number of observed cases, national estimate could not be accurately derived.
