# Supplementary material for: A Review of Sports-Related, Life-Threatening Injuries Presenting to Emergency Departments, 2009–18
Source: West J Emerg Med. 2025 Feb 24;26(3):627–31. doi: 10.5811/westjem.18630 (PMC12208023; doi:10.5811/westjem.18630)
Supplement: Supplementary file 1 [file wjem-26-627-s001.docx]

**Appendix 1.** Sports Related External Cause of Injury Codes

Sports or athletic activity ICD-9 code ICD 10-code

| Accident caused by air gun | E922.4 | W34.010, W34.110 |
| --- | --- | --- |
| Accident caused by paintball gun | E922.5 | W34.011, W34.111 |
| Accident due to diving or jumping into water | E883.0 | W16 (.0, .1, .3, .4, .5, .6, .7, .8, .9), W22.04 |
| Accident due to change in air pressure due to diving | E902.2 | W94.21 |
| Accident involving motor-driven snow vehicle | E820 (.0, .1, .5, .6, .8, .9) | V86 (.52, .62, .92) |
| Accident involving other off-road motor vehicle | E821 (.0, .1, .5, .6, .8, .9) | V86 (.53, .55, .56, .59, .63, .65, .66, .69, .93, .95, .96, .966) |
| Accident involving ridden animal or animal drawn vehicle | E822.5, E823.5, E824.5, E825.5, E827 (.2, .8, .9), E828 (.0, .2, .8, .9) | V80 (.01, .11, .31, .41, .51, .61, .71, .81), V80.2, V80 (.918, .919) |
| Accident occurring in place for recreation and sport | E849.4 | Y92.3, Y92.482, Y92.83 |
| Accidental drowning and submersion during sport or recreation | E910.1, E910.2 | W69, V90 (.05, .08), V91.25 |
| Accidental drowning during waterskiing | E910.0 | Y93.17, V90.07 |
| Accidental fall from playground equipment | E884.0 | W09 |
| Activities involving walking and running | E001 | Y93.0 |
| Activities involving water and watercraft | E002 | Y93.1 |
| Activities involving snow and ice | E003 | Y93.2 |
| Activities involving climbing, rappelling and jumping off | E004 | Y93.3 |
| Activities involving dancing and other rhythmic movement | E005 | Y93.4 |
| Activities involving other sports and athletics played individually | E006 | Y93.5 |
| Activities involving other sports and athletics played as a team or group | E007 | Y93.6 |
| Activities involving other specified sports and athletics | E008 | Y93.7 |
| Activity involving other cardiorespiratory exercise ` | E009 | Y93.A |
| Activity involving other muscle strengthening exercises | E010 | Y93.B |
| Pedal cycling | E800.3, E 801.3, E 802.3, E 803.3, E804.3, E805.3, E806.3, E807.3, E810.6, E811.6, E812.6, E813.6, E814.6, E815.6, E816.6, E817.6, E818.6, E819.6, E822.2, E823.6, E824.6, E825.6, E826 (.1,.2,.8,.9) | V10-V19 |
| Fall from nonmotorized scooter | E885.0 | V00.14 |
| Fall from roller skates/inline skates | E885.1 | V00 (.1, .2), V01 (.01, .11, .91), V02 (.01, .11, .91), V03 (.01, .11, .91), V04 (.01, .11, .91), V05 (.01, .11, .91), V06 (.01, .11, .91) |
| Fall from skateboard | E885.2 | V00.13, V01 (.02, .12, .92), V02 (.02, .12, .92), V03 (.02, .12, .92), V04 (.02, .12, .92), V05 (.02, .12, .92), V06 (.02, .12, .92) |
| Fall from skiing | E885.3 | V00.32 |
| Fall from snowboard | E885.4 | V00.31 |
| Fall on same level from collision, pushing or shoving, by or with other person in sports | E886.0 | W03 |
| Striking in sports | E917.0, E917.5 | W21 |
| Overexertion | E927 | X50.0 |
| Hang gliding injury | E842 | V96.11 |
| Heat stroke | E900.9 | X30 |
